# Metabolomic Profiling of Wildtype and Transgenic *Giardia lamblia* Strains by ^1^H HR-MAS NMR Spectroscopy

**DOI:** 10.3390/metabo10020053

**Published:** 2020-01-30

**Authors:** Joachim Müller, Martina Vermathen, David Leitsch, Peter Vermathen, Norbert Müller

**Affiliations:** 1 Institute of Parasitology, Vetsuisse Faculty, University of Bern, Länggass-Strasse 122, CH-3012 Bern, Switzerland; norbert.mueller@vetsuisse.unibe.ch; 2 Department of Chemistry and Biochemistry, University of Bern, Freiestrasse 3, CH-3012 Bern, Switzerland; martina.vermathen@dcb.unibe.ch; 3 Institute of Specific Prophylaxis and Tropical Medicine, Medical University of Vienna, Kinderspitalgasse 15, A-1090 Vienna, Austria; david.leitsch@meduniwien.ac.at; 4 Departments of BioMedical Research and Radiology, University and Inselspital Bern, sitem-insel AG Freiburgstr. 3, CH-3010 Bern, Switzerland; peter.vermathen@insel.ch

**Keywords:** amino acids, arginine, metabolism, giardia, HR-MAS, resistance

## Abstract

*Giardia lamblia*, a causative agent of persistent diarrhea in humans, domestic animals, and cattle, is usually treated with nitro compounds. Consequently, enzymes involved in anaerobic nitro reduction have been investigated in detail as potential targets. Their role within the normal metabolic context is, however, not understood. Using ^1^H high-resolution magic angle spinning (HR-MAS) NMR spectroscopy, we analyzed the metabolomes of *G. lamblia* trophozoites overexpressing three nitroreductases (NR1–NR3) and thioredoxin reductase (TrxR), most likely a scavenger of reactive oxygen species, as suggested by the results published in this study. We compared the patterns to convenient controls and to the situation in the nitro drug resistant strain C4 where NR1 is downregulated. We identified 27 metabolites in *G. lamblia* trophozoites. Excluding metabolites of high variability among different wildtype populations, only trophozoites overexpressing NR1 presented a distinct pattern of nine metabolites, in particular arginine catabolites, differing from the respective controls. This pattern matched a differential pattern between wildtype and strain C4. This suggests that NR1 interferes with arginine and thus energy metabolism. The exact metabolic function of NR1 (and the other nitroreductases) remains to be elucidated.

## 1. Introduction

*Giardia lamblia* is an anaerobic or microaerophilic unicellular diplomonad causing persistent diarrhea in humans and various domestic animals. The life cycle comprises two stages, namely, the pear-shaped, flagellated trophozoite proliferating in the upper part of the small intestine, and the non-proliferative cyst representing the infectious stage of the parasite [1,2].

Analysis of the genome [3] has revealed prokaryote-like features such as short promoter sequences, a nearly complete absence of introns, and high similarities of some key enzymes of energy and intermediate metabolism, with prokaryotic enzymes most likely acquired by lateral transfer [4,5]. Thus, it is not surprising that the metabolism of *G. lamblia* trophozoites has unusual features in comparison to host cells. The anaerobic energy metabolism [6] is chemoheterotrophic yielding ethanol, acetic acid, and alanine as fermentation end products. Moreover, arginine is used as the main energy supply via arginine deiminase (ADI), ornithine carbamoyl transferase, and carbamate kinase, as detailed in earlier studies [7,8,9].

The treatment against giardiasis comprises nitro compounds such as metronidazole (MET), other 5-nitroimidazole compounds, or nitazoxanide (NTZ) [10,11]. Moreover, *G. lamblia* is susceptible to a variety of antibiotics because of its prokaryote-like transcription and translation machineries, as well as to albendazole [12]. According to a commonly accepted model, nitro compounds are activated by reduction yielding toxic intermediates causing nitrosative stress [13,14]. Pyruvate ferredoxin oxidoreductase (PFOR) is a main supplier of electrons [6,15]. This explains why knock-down of this enzyme is correlated with increased resistance to MET [16]. Studies with resistant strains have revealed, however, that resistance does not always correlate with reduced PFOR activity [17,18]. Other enzymes potentially involved in the reduction of nitro drugs are the nitroreductases (NR)1 [19,20] and NR2 [21,22]. Recently, a third homologue of NR1 and NR2 with weak nitroreductase activity, NR3, has been described [23]. All three NRs have higher quinone reductase than nitroreductase activities in vitro [22,23]. Overexpression of NR1 results in an increased susceptibility to nitro compounds in *Escherichia coli* [19,22,23] and in *G. lamblia* [19,23], whereas overexpression of NR2 results in an increased resistance in *E. coli* [21,22,23] in independent studies. Their function within the intermediary metabolism under physiological conditions (i.e., in the absence of nitro drugs) is completely unknown.

Thioredoxin-reductase (TrxR) has nitroreductase activity in vitro [15]. Overexpression of the wildtype allele in the *G. lamblia* strain WBC6 does not alter the susceptibility to NTZ and only slightly alters the susceptibility to MET, and overexpression of a dominant negative mutant allele has no effects on drug resistance [24]. In resistant strains of a different genetic background, however, TrxR is overexpressed [25]. Specific functions are not known, to date.

In order to get an insight into potential functions of these enzymes, the first step would be to compare metabolomic patterns of strains with altered expression levels of the respective genes. In a previous study, we introduced high-resolution magic angle spinning (HR-MAS) NMR spectroscopy as a suitable tool to study the metabolome of *G. lamblia* [26]. HR-MAS yields well-resolved liquid-like ^1^H-NMR spectra from semi-solid materials, reducing resonance line broadening effects caused by dipolar interactions and magnetic susceptibility differences inherent to most semi-solid materials [27]. Therefore, HR-MAS NMR has become a very attractive tool in the analysis of small metabolites from biological materials such as cells and tissue samples [28,29] or food samples [30,31,32]. Moreover, this method has proven to be suitable for the determination of biomarkers for the metabolic response to anticancer drugs [33,34,35].

Here, we present a metabolomic study using ^1^H HR-MAS NMR spectroscopy comparing transgenic *G. lamblia* strains overexpressing genes encoding for thioredoxin reductase and nitroreductases. Unfortunately, knock-down of the expression of the respective genes is not possible in our hands. An exception is TrxR, where a dominant negative mutant allele exists, the gene product of which forms an inactive complex with the wildtype TrxR. Another exception is the nitro drug resistant strain *G. lamblia* C4 derived from the wildtype WBC6 [17], which has significantly reduced NR1 protein levels when grown in the presence of NTZ [19,25].

In order to identify potential metabolomic patterns related to the overexpression of the respective genes, we eliminated metabolites from the discussion that showed strong differences between independent wildtype populations. Moreover, as we identified a distinct pattern in NR1 overexpressors only, we tested the observed pattern by a comparison between the wildtype WBC6 and the derived nitro drug-resistant strain C4. The strains used in this study are listed in Table 1.

## 2. Results and Discussion

### 2.1. Relative Amounts of Identified Metabolites

Using ^1^H-HR-MAS NMR, we identified 27 metabolites in *G. lamblia* WBC6 trophozoites, as listed in Table 2. The values of all individual integrals are available in the Supplementary Materials section (Table S1). In order to discriminate “natural” metabolite differences between wildtype populations from those related to transgenic modifications, we compared, in a first step, the metabolomes of two completely independent populations of WBC6 wildtype trophozoites (Table 2).

Nine of the metabolites identified, namely, glucose-1-P (Glc-1-P), seven amino acids (alanine, asparagine, cysteine, glutamine, leucine, threonine, valine), and trimethylamine (TMA) were significantly different between independent populations of WBC6 wildtype populations (Table 2, columns on the left). The variability of these nine metabolites may be due to rapid turn-over rates due to their pivotal roles in energy and intermediary metabolism. Furthermore, the variability may reflect the potential of the *Giardia* strain WBC6 to adapt to changing environmental conditions. Consequently, in order to identify patterns discriminating between strains, these metabolites had to be excluded. For an overview, principal component analysis (PCA) and orthogonal partial least squares discriminant analysis (oPLS-DA) plots containing all *Giardia* strains given in Table 2 are displayed in Figure S1.

### 2.2. Phenotype and Metabolite Patterns Related to Thioredoxin Reductase Overexpression

**Figure 1:**
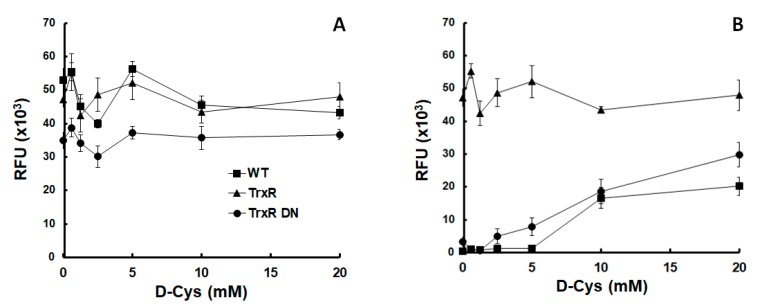
Thioredoxin reductase protected *Giardia lamblia* trophozoites from oxygen. Growth of *G. lamblia* wildtype trophozoites (WT) and of trophozoites overexpressing either wildtype thioredoxin reductase (TrxR) or a dominant negative mutant allele (TrxR DN) under anaerobic (**A**) or semi-aerobic (**B**) conditions in medium supplemented with different amounts of D-cysteine (D-Cys).

Because overexpression of either allele of TrxR had little effect on nitro drug susceptibility, we investigated whether oxygen differentially affected the growth of strains overexpressing either the wildtype allele (TrxR) or a dominant negative allele (TrxR DN). There was no marked difference between the three strains tested under anaerobic growth conditions (Figure 1A). Under semi-aerobic conditions, however, the untransformed wildtype strain grew only in the presence of more than 10 mM D-cysteine (D-Cys) added to the medium. The strain overexpressing the dominant negative TrxR allele started growing already at 2.5 mM D-Cys. The strain overexpressing the wildtype TrxR grew even in the absence of D-Cys, reaching higher densities after 3 days than the two other strains (Figure 1B).

Our results suggest that TrxR acts as a scavenger of reactive oxygen species, as suggested by previous studies [5,36]. Because D-Cys is non-proteinogenic, we attribute the protective effect to a direct inactivation of oxygen, not to an indirect effect such as an increased synthesis of Cys-rich proteins. The TrxR DN strain may need less D-Cys than the wildtype trophozoites (WT) to resume growth because the protein itself may act as a scavenger independently of enzyme activities.

**Figure 2:**
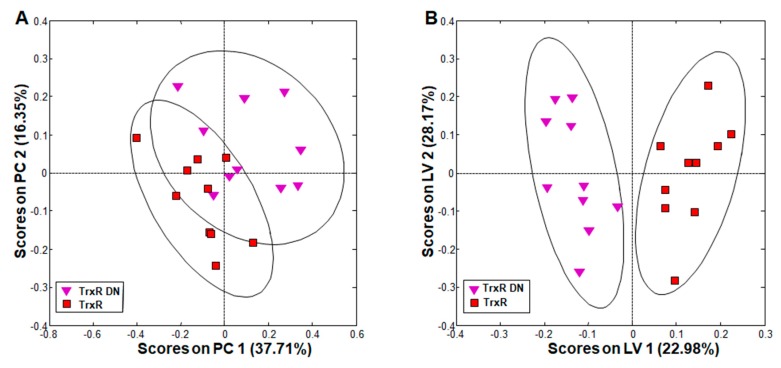
Principal component analysis (PCA) and orthogonal partial least squares discriminant analysis (oPLS-DA) of all integral regions (138 buckets) obtained by ^1^H high-resolution magic angle spinning (HR-MAS) NMR analysis of TrxR and TrxR DN trophozoites. The ellipses show 83.4% confidence intervals. The strains are detailed in Table 1. (**A**) PCA; (**B**) oPLS-DA. *R*^2^ and *Q*^2^ values for the oPLS model are given in Table S2. PC: principal component; LV: latent variable.

The comparison of metabolite patterns in *G. lamblia* trophozoites overexpressing either the wildtype thioredoxin reductase (TrxR) or a dominant negative allele (TrxR DN) revealed clear clustering of each group in principal component analysis (PCA; Figure 2A). Orthogonal partial least squares discriminant analysis (oPLS-DA) showed complete and significant separation between the groups along the first, predictive PLS component latent variable (LV) 1 (Figure 2B).

A detailed comparison of single metabolites revealed that these differences could be attributed to acetate, which was significantly increased, and lysine, which was significantly decreased in TrxR DN as compared to TrxR (Table 2, columns 3 and 4). Other separating contributions only derived from metabolites with pool sizes varying between wildtype populations. Because acetate is an indirect product of PFOR, overexpressors of the wildtype allele may reduce it more efficiently to ethanol than the overexpressors of the dominant negative mutant allele. As the catabolism of lysine yields acetyl-coenzymeA (and thus acetate) as a final product (see [37] and references therein), the increase of acetate may also be directly linked to an increased catabolism of lysine, thereby causing the observed changes in the pool sizes of both metabolites.

### 2.3. Metabolite Patterns Related to Nitroreductase Overexpression

**Figure 3:**
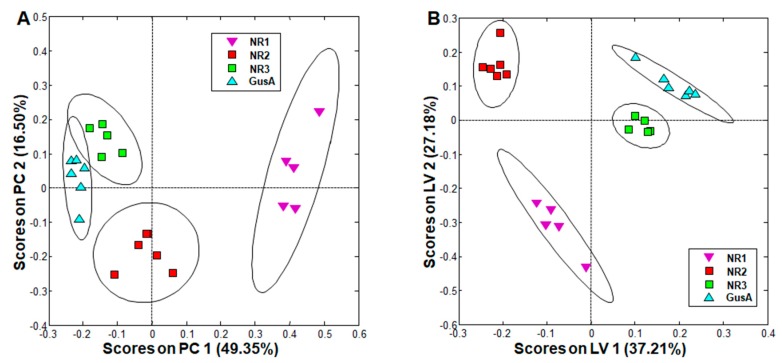
PCA and oPLS-DA of all integral regions (138 buckets) obtained by ^1^H-HR-MAS NMR analysis of GusA, NR1, NR2, and NR3 trophozoites. The ellipses show 83.4% confidence intervals. *R*^2^ and *Q*^2^ values for the oPLS model are given in Table S2. The strains are detailed in Table 1. (**A**) PCA; (**B**) oPLS-DA.

When comparing trophozoites overexpressing the nitro reductases NR1, NR2, and NR3 to a control strain overexpressing glucuronidase A (see Table 1 for phenotypes), PCA resulted in complete separation of all classes, and oPLS-DA revealed significant differences between all these strains (Figure 3). More specifically, the NR3 and GusA groups were clustered closer together, whereas the NR1 group was the furthest away and was already distinguished in PCA from all other groups solely by its positive scores along principal component (PC) 1 (Figure 3A).

Upon exclusion of metabolites differing in wildtype population, the most striking differences were observed in NR1 overexpressing trophozoites, with nine metabolites significantly changed as compared to the GusA control, namely, acetate, glutamine, phenylalanine, and tyrosine with significantly lower levels, and cystine, methionine, ornithine, citrulline, and pipecolic acid with significantly higher levels in NR1 overexpressors as compared to GusA control. Acetate was significantly decreased in all NR overexpressors as compared to the GusA control (Table 2, columns on the right). In the strain overexpressing NR2, besides acetate, only ornithine and cysteine followed the same pattern as in the NR1 overexpressor. In the strain overexpressing NR3, only acetate was significantly affected.

In particular, amino acids generated during the catabolism of the basic amino acids arginine (ornithine and citrulline) and lysine (pipecolic acid) were significantly increased in NR1 expressing trophozoites as compared to the GusA control (Table 2).

**Figure 4:**
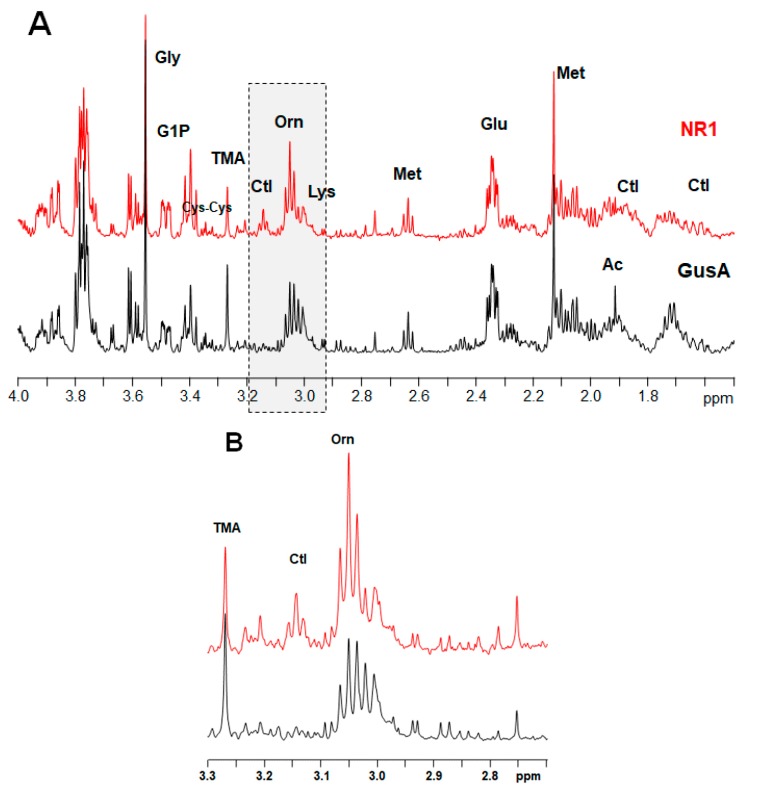
Representative ^1^H HR-MAS NMR spectra of suspensions of NR1 and GusA trophozoites in phosphate buffered saline (PBS). The resonances of citrulline (Ctl) and Ornithine (Orn) between 3.0 and 3.2 ppm are highlighted by the gray box. (**A**) Spectral region from 1.5 ppm to 4 ppm, (**B**) highlighted region in detail.

A typical NMR spectrum representing peaks for ornithine and citrulline from NR1 and GusA trophozoites is shown in Figure 4.

Thus, taken together, we could identify a distinct pattern of nine metabolites differential between NR1 and GusA control trophozoites.

### 2.4. Comparison of the NR1 Pattern with the Situation in Nitro Drug-Resistant Trophozoites

These findings prompted us to test whether the pattern obtained by comparing NR1 overexpressing trophozoites (with an increased susceptibility to NTZ) to GusA control trophozoites could be linked to the nitro drug susceptibility. For this, we compared the metabolomes of WBC6 wildtype trophozoites with trophozoites of the nitro drug-resistant strain C4 with reduced NR1 activity. Individual normalized peak integrals and a comparison of all metabolites between WBC6 and C4 trophozoites are given in the Supplementary Materials section (Table S3 and Figure S2).

**Figure 5:**
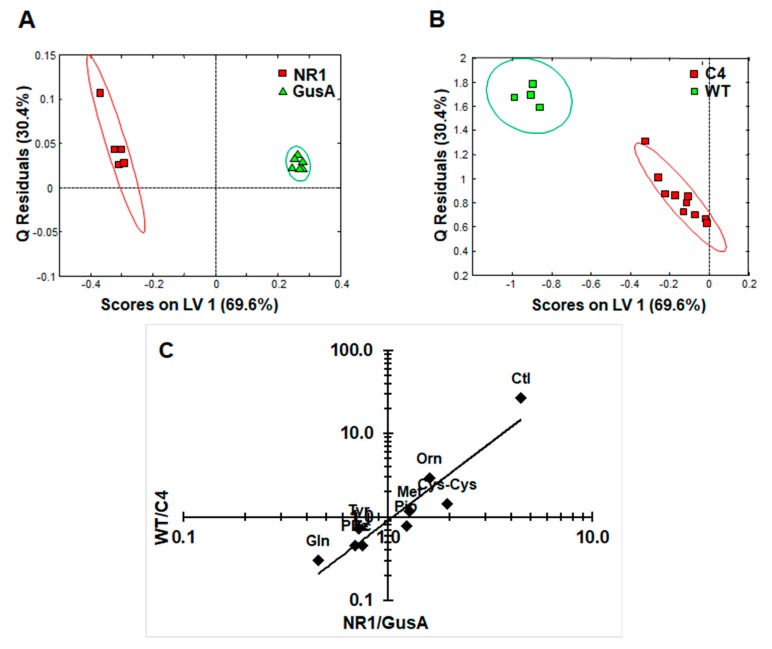
The metabolic pattern obtained by comparing NR1 versus GusA can be linked to the nitro drug susceptibility. (**A**) oPLS-DA score plots displaying the model calculated for distinguishing NR1 from GusA along the latent variable LV 1. (**B**) Class prediction based on the model shown in (**A**) applied to C4 and WT strains. The ellipses show 83.4% confidence intervals. (**C**) Ratios of the peak integrals of the eight differential metabolites listed above from NR1/GusA vs. WT/C4. The correlation between metabolite ratios in *G. lamblia* NR1 vs. GusA and WT vs. C4 trophozoites (see Table 1) was highly significant (Pearsons correlation coefficient 0.942; *p* < 0.0005). *R*^2^ and *Q*^2^ values for the oPLS model are given in Table S2.

Subsequently, an oPLS-DA model calculated for discrimination between NR1 and GusA (Figure 5A) was applied to the nitro drug-resistant C4 trophozoites and wildtype trophozoites (WT), as shown in Figure 5B. The predictive value of the model led to complete separation of C4 versus WT, that is, suggesting a correlation between nitroreductase activity and the metabolome. In a next step, we plotted the relative changes observed in NR1 compared to GusA of the nine differential metabolites listed above versus the corresponding relative changes obtained in WT compared to C4 (Figure 5C).

As shown in Figure 5C, the pattern of the nine identified metabolites inversely correlated with a pattern of the same metabolites in the nitro drug-resistant strain C4, which has lower NR1 levels than its corresponding wildtype. The correlation was highly significant.

The catabolism of arginine, a major energy source for *G. lamblia* [8,26], is of particular interest within this context. Citrulline and ornithine, the first and the second catabolites of arginine, are positively correlated with NR1 levels, whereas glutamine, an indirect catabolite of ornithine [38], is negatively correlated with NR1 levels. This suggests that NR1 interferes with the catabolism of arginine. Because the ATP levels are unaltered in NR1, as well as in all other transgenic trophozoites, a direct interference with energy supply is unlikely. In *G. lamblia*, arginine catabolism is not only a major source for ATP, but has also other functions. Within this context, arginine deiminase (ADI) is of particular interest. ADI does not only transform free arginine into citrulline [39], but also arginine residues in a variety of proteins including variant surface proteins [40]. Accumulation of inhibitory amounts of citrulline of this enzyme may thus affect regulatory pathways depending on ADI, a “moonlighting” protein, as evidenced by recent studies [41,42]. Because NR1 overexpressed and purified from *E. coli* has a much higher quinone than nitroreductase activity in vitro [22,23], it is possible that it interferes with susceptibility/resistance to nitro compounds, not (only) by direct reduction of these compounds, but rather indirectly via reduction of an unknown quinone substrate or by interfering with coenzymes involved in electron transfer [18]. The exact function of this enzyme remains to be elucidated.

## 3. Conclusions

In contrast to other organisms, the metabolome plasticity of the early diverging eukaryont *G. lamblia* is far from being elucidated, especially in response to genetic transformation and/or drug-based selection. The present study could therefore be regarded as a paradigm extendable to studies on other extracellular parasites such as, for example, amebae [2], trichomonads, or even helminths [43]. These investigations may yield novel antiparasitic drug targets.

## 4. Materials and Methods

### 4.1. Biochemicals

If not otherwise stated, all biochemical reagents were from Sigma (St Louis, MO, USA). D_2_O-based 50 mM phosphate-buffered saline (PBS) was prepared from mixing adequate amounts of 50 mM K_2_HPO_4_ and NaH_2_PO_4_ solutions in D_2_O containing 0.9% NaCl to give a pH of 7.0 (corresponding to a pD of 7.4).

### 4.2. Growth of Thioredoxin Reductase Overexpressors

*G. lamblia* trophozoites overexpressing a wildtype allele of thioredoxin reductase (TrxR), a dominant negative mutant allele (TrxR DN; see [24]), or wildtype trophozoites were seeded into 96-well plates (10^3^ trophozoites per well) containing normal culture medium or medium supplemented with increasing amounts of D-cysteine. D-cysteine was used instead of L-cysteine because it is not proteinogenic. The plates were incubated under anaerobic (85% N_2_, 10% H_2_, 5% CO_2_) or semi-aerobic (85% N_2_, 10% CO_2_, 5% O_2_) conditions. After 3 days, cells were quantified using the Alamar blue assay, as described elsewhere [44,45]. The assays were run in quadruplicate.

### 4.3. Preparation of Cell Samples for HR-MAS NMR

*G. lamblia* trophozoites from the strains listed in Table 1 were grown to confluence in modified TYI-S33 medium and harvested by chilling on ice, as previously described [18,23,24]. After centrifugation, trophozoites were washed once in ice-cold PBS, followed by two washes with ice-cold D_2_O-based PBS, counted in a Neubauer chamber, and shock frozen in liquid nitrogen. All samples were stored in aliquots corresponding to 3 × 10^6^ cells at −70 °C until further analysis [26]. The nitroreductase overexpressors were compared to a GusA overexpressor and not directly to an untransfected wildtype strain because all strains had to undergo a selection procedure of transfectants, which was shown to influence expression of various genes [46].

### 4.4. HR-MAS NMR Spectroscopy and Processing of Spectra

^1^H HR-MAS NMR experiments of cell samples were performed on a 500 MHz Bruker Avance II spectrometer (Bruker BioSpin, Fällanden, Switzerland) with a 4 mm HR-MAS dual inverse ^1^H/^13^C probe equipped with a z-gradient directed along the magic angle axis. Each sample was thawed during 2 min immediately before HR-MAS NMR measurement. Suspensions corresponding to 3 × 10^6^ cells were filled into ZrO_2_-HR-MAS rotors with PTFE (Teflon) inserts providing a sample volume of 50 μL. The sample amount inside the rotor was determined by weight control. The rotor was spun at a MAS rate of 3 kHz during measurements. The temperature was set to 279 K (nominal), allowing the sample to equilibrate during 5 min. ^1^H spectra were recorded using a 1D PROJECT (periodic refocusing of J evolution by coherence transfer) pulse sequence with water pre-saturation and a rotor-synchronized T_2_ filter of 115 ms to suppress broad components with short T_2_ relaxation times. Each spectrum was acquired with 1024 scans, a spectral width of 6010 Hz, a data size of 16 k points, an acquisition time of 1.36 s, and a relaxation delay of 4 s. ^1^H NMR resonance assignments were based on the spectral analysis previously published [26]. To confirm resonance assignments, additional 2D ^1^H^1^H-TOCSY spectra were acquired for at least one sample within each sample group using the DIPSI2 sequence (“dipsi2phpr” from the Bruker pulse program library) with water pre-saturation during relaxation delay.

Data acquisition and spectral processing were performed with the Bruker TopSpin v 3.2 Pl5 software. All spectra were processed identically—the summed free induction decays (FIDs) were exponentially multiplied with a line broadening factor of 1 Hz and Fourier transformed. The resulting spectra were phased and calibrated to the γ-CH_3_ resonance of threonine (Thr) at 1.326 ppm. Baseline correction for the spectral region of 0–5.7 ppm was performed by applying a cubic spline function with individually set baseline points. The spectral region between 5.7 and 10 ppm was baseline-corrected using the underground removal tool of the Bruker AMIX 3.9.14 software applying a filter of 30 Hz.

### 4.5. Bioinformatics and Statistical Methods

Statistical analysis of the inhibition tests was performed with suitable tools from the open source software package R [47]. The results were compared to the controls separately for each strain and analysed by two-sided paired *t*-tests. The *p*-values were corrected for multiple comparisons.

Multivariate statistical data analysis was performed using MATLAB R2012a (Mathworks) and PLS-Toolbox 7.5.2 (Eigenvector Research, Inc., Manson, WA, USA). The processed NMR spectra were subdivided into 138 individually sized buckets (integral regions) to cover single resonances as much as possible in the spectral range between 0 and 10 ppm. Bucket selection was guided by an overlay of summed spectra from each sample group. The spectral region of the residual water resonance (4.66–5.2 ppm) and noise regions were excluded. Both bucket selection and integration of bucket regions were performed with the Bruker TopSpin v 3.2 Pl5 software. The numeric bucket integrals were exported into the Excel (Microsoft Office Professional Plus 2016) software, and the buckets were assigned as far as possible to contributing metabolites. The subsequent steps were performed using the PLS-Toolbox software. Probabilistic quotient normalization (PQN) was applied to the integral regions to account for different sample amounts [48]. Mean centering was applied to the buckets, as well as pareto scaling to reduce the relative importance of large values [49]. The samples (spectra) were grouped according to the corresponding *G. lamblia* strain, as given in Table 2, and the number of samples per group was *n* = 4–10 (Table 2). Principal component analysis (PCA) was applied to probe for clustering. The first two principal components (PC 1, PC 2) were plotted to generate the PC score plots to visualize unsupervised variation of the datasets within each group. For testing, if the groups can be distinguished on the basis of their metabolite spectra, orthogonal partial least squares discriminant analysis (oPLS-DA) was performed using sample group assignment as *y*-variables. One to three latent variables (LV) were calculated, and LV1 and LV2 were plotted to obtain the oPLS-score plots. When only one LV was calculated (as suggested by the software in order not to overfit the data), this value was plotted against the Q residuals. For both PCA and oPLS, 83.4% confidence ellipses, indicating a statistical significant separation of *p* < 0.05 when not overlapping, were superimposed on the score plots to illustrate the goodness of class separation [50]. The venetian blinds method was used for cross validation of the oPLS models. Parameters of the oPLS including the goodness of fit (*R*^2^) and the predictive ability (*Q*^2^) are summarized in Table S2.

For univariate statistical analysis, integrals of representative spectral regions for each of the 27 metabolites were evaluated. These integrals were selected such that either single or averages of multiple isolated resonances from each metabolite were included, minimizing contributions from other metabolite resonances as much as possible. The selection of integrals is visualized in Figure S3 on representative spectra. The resonance integrals or corresponding buckets were extracted from the PQN-normalized data matrix that was created for multivariate statistics, as described above. For each integral, the mean value and standard deviation was calculated using the Excel software. Single factor ANOVA and *t*-test (Excel) were used to probe for metabolite differences between the *G. lamblia* strains. All *p*-values were multiplied with a factor of 27 to correct for multiple comparisons (Bonferroni correction).

## Figures and Tables

**Table 1:** List of the transgenic *Giardia lamblia* strains used in this study. All transgenes were fused to the strong, constitutive arginine deiminase (ADI)-promotor. CDS, coding sequence; NR, nitroreductase; TrxR, thioredoxin reductase; TrxR DN, dominant negative mutation of TrxR. Please note that we have kept our original designations NR1, NR2, and NR3 to be in frame with our previous publications.

Strain	Characteristics	Accession	References
WBC6	Wildtype strain (WT)	No transgene	
C4	Nitro drug-resistant strain derived from WT	No transgene	
GusA	WT overexpressing *E. coli* glucuronidase A	*E. coli* K-12 CDS 1785 074 –1785 074	
NR1	WT overexpressing *G. lamblia* Fd-NR2 (“NR1”) Higher susceptibility to nitro drugs in *G. lamblia* and *E. coli*	Giardia DB 22677	[19,23]
NR2	WT overexpressing *G. lamblia* Fd-NR1 (“NR2”) Better resistance to nitro drugs in *E. coli*	Giardia DB 6175	[21,23]
NR3	WT overexpressing *G. lamblia* NR family protein (“NR3”)	Giardia DB 15307	[23]
TrxR	WT overexpressing *G. lamblia* TrxR wildtype	Giardia DB 9827	[24]
TrxR DN	WT overexpressing *G. lamblia* TrxR-dominant negative allele Higher susceptibility to oxygen	Giardia DB 9827	[24] This work

**Table 2:** Metabolites identified in *G. lamblia* WBC6 wildtype trophozoites (WT; two independent experiments), in trophozoites overexpressing thioredoxin reductase (TrxR) or a dominant negative mutation of TrxR (TrxR DN) as a control, and in trophozoites overexpressing the nitroreductases NR1, NR2, NR3, or GusA as a control. The values represent normalized integrated peak areas obtained by integration of ^1^H-NMR resonances. Values are given as mean values ± SD of arbitrary units for *n* replicates as indicated. Metabolites with significant differences from the respective controls (i.e., TrxR DN for TrxR and GusA for the NRs) after Bonferroni correction for multiple comparisons are printed in bold (ANOVA followed by *t*-tests; +, *p* < 0.05; °, *p* < 0.01; *, *p* < 0.001). TMA: trimethylamine.

Metabolite	WT 1 *n* = 10	WT 2 *n* = 4	TrxR *n* = 10	TrxR_DN *n* = 10	GusA *n* = 6	NR1 *n* = 5	NR2 *n* = 6	NR3 *n* = 5
*Carbohydrates and related*								
Acetate	0.77 ± 0.05	0.85 ± 0.03	0.57 ± 0.03	0.65 ± 0.04 *	0.65 ± 0.02	0.49 ± 0.02 *	**0.58 ± 0.02 ^+^**	**0.58 ± 0.02 ^+^**
Citrate	0.17 ± 0.07	0.20 ± 0.01	0.18 ± 0.09	0.24 ± 0.06	0.11 ± 0.05	0.32 ± 0.10	0.18 ± 0.08	0.16 ± 0.05
Glucose-1-phosphate	4.46 ± 0.65	1.65 ± 0.1 *	4.59 ± 0.40	5.16 ± 0.66	1.69 ± 0.19	2.31 ± 0.13 °	1.71 ± 0.24	1.88 ± 0.14
*Amino acids*								
Alanine	6.02 ± 0.77	7.63 ± 0.33 *	4.88 ± 0.88	5.20 ± 0.79	6.95 ± 0.30	5.40 ± 0.25	6.00 ± 0.48	6.80 ± 0.36
Asparagine	0.18 ± 0.02	0.26 ± 0.03 *	0.19 ± 0.04	0.22 ± 0.03	0.22 ± 0.02	0.18 ± 0.07	0.21 ± 0.03	0.26 ± 0.02
Citrulline	0.19 ± 0.09	0.26 ± 0.09	0.14 ± 0.11	0.18 ± 0.05	0.26 ± 0.07	1.16 ± 0.14 *	0.46 ± 0.18	0.24 ± 0.13
Cysteine	0.56 ± 0.12	0.29 ± 0.07 ^+^	0.40 ± 0.13	0.32 ± 0.04	0.22 ± 0.09	0.38 ± 0.07	0.29 ± 0.15	0.29 ± 0.14
Cystine (Cys-Cys)	0.12 ± 0.04	0.13 ± 0.03	0.12 ± 0.05	0.15 ± 0.04	0.24 ± 0.05	**0.47 ± 0.04 ^+^**	**0.42 ± 0.06 ^+^**	0.27 ± 0.04
Glutamate	7.03 ± 0.28	4.94 ± 0.31 *	7.27 ± 0.25	7.12 ± 0.24	5.32 ± 0.15	5.35 ± 0.15	5.14 ± 0.12	5.41 ± 0.20
Glutamine	0.48 ± 0.12	0.72 ± 0.24	0.45 ± 0.14	0.48 ± 0.09	0.94 ± 0.10	0.34 ± 0.09 *	0.66 ± 0.32	0.91 ± 0.11
Glycine	2.18 ± 0.10	2.85 ± 0.03	2.38 ± 0.09	2.21 ± 0.12	2.86 ± 0.06	2.40 ± 0.11	2.65 ± 0.03	2.96 ± 0.07
Histidine	0.19 ± 0.04	0.28 ± 0.02	0.14 ± 0.04	0.16 ± 0.04	0.26 ± 0.02	0.14 ± 0.05	0.21 ± 0.03	0.22 ± 0.04
Isoleucine	0.42 ± 0.18	0.68 ± 0.15	0.35 ± 0.10	0.35 ± 0.10	0.72 ± 0.12	0.68 ± 0.04	0.88 ± 0.11	0.77 ± 0.08
Leucine	4.27 ± 0.18	5.89 ± 0.10 *	3.92 ± 0.16	3.76 ± 0.17	5.95 ± 0.06	4.09 ± 0.18	5.52 ± 0.15	5.19 ± 0.14
Lysine	4.67 ± 0.36	4.26 ± 0.18	5.45 ± 0.24	**5.02 ± 0.24 °**	4.37 ± 0.27	4.68 ± 0.18	4.08 ± 0.24	4.33 ± 0.25
Methionine	0.95 ± 0.08	1.06 ± 0.07	1.02 ± 0.05	0.98 ± 0.05	1.02 ± 0.05	**1.30 ± 0.04 °**	1.15 ± 0.05	1.09 ± 0.05
Ornithine	1.84 ± 0.13	1.71 ± 0.03	1.90 ± 0.11	1.72 ± 0.13	1.66 ± 0.02	2.65 ± 0.17 *	**1.92 ± 0.12 °**	1.81 ± 0.08
Phenylalanine	0.30 ± 0.02	0.33 ± 0.02	0.28 ± 0.02	0.27 ± 0.04	0.29 ± 0.01	**0.20 ± 0.04 ^+^**	0.25 ± 0.04	0.28 ± 0.02
Pipecolic acid	0.64 ± 0.08	0.69 ± 0.12	0.73 ± 0.08	0.72 ± 0.11	1.00 ± 0.04	1.23 ± 0.06 *	1.07 ± 0.07	1.00 ± 0.10
Proline	0.56 ± 0.12	0.40 ± 0.22	0.50 ± 0.12	0.50 ± 0.12	0.68 ± 0.08	0.54 ± 0.05	0.64 ± 0.17	0.66 ± 0.09
Threonine	2.96 ± 0.12	2.61 ± 0.07 ^+^	3.27 ± 0.12	3.03 ± 0.14	2.75 ± 0.11	2.50 ± 0.07	2.83 ± 0.14	3.00 ± 0.07
Tryptophan	0.11 ± 0.03	0.07 ± 0.03	0.09 ± 0.02	0.10 ± 0.03	0.05 ± 0.02	0.04 ± 0.02	0.08 ± 0.04	0.03 ± 0.01
Tyrosine	0.38 ± 0.04	0.37 ± 0.02	0.37 ± 0.02	0.34 ± 0.02	0.36 ± 0.02	0.26 ± 0.02 *	0.32 ± 0.07	0.36 ± 0.02
Valine	4.24 ± 0.19	5.85 ± 0.08 *	4.72 ± 0.20	4.46 ± 0.26	5.87 ± 0.13	5.27 ± 0.19	5.94 ± 0.25	5.93 ± 0.16
*Cofactors*								
Adenosine triphosphate	0.25 ± 0.03	0.22 ± 0.02	0.27 ± 0.04	0.25 ± 0.03	0.24 ± 0.02	0.26 ± 0.02	0.30 ± 0.05	0.25 ± 0.02
Nicotinamide-adenine-dinucleotide	0.08 ± 0.03	0.06 ± 0.01	0.08 ± 0.01	0.08 ± 0.02	0.07 ± 0.03	0.07 ± 0.02	0.08 ± 0.03	0.08 ± 0.01
*Other*								
Trimethylamine	1.18 ± 0.04	1.09 ± 0.02 ^+^	1.16 ± 0.06	1.16 ± 0.06	1.12 ± 0.03	0.71 ± 0.03 *	**0.87 ± 0.08 °**	1.04 ± 0.03

## Supplementary Materials

The following are available online at https://www.mdpi.com/2218-1989/10/2/53/s1, Figure S1: PCA and oPLS-DA of all integral regions (138 buckets) obtained by ^1^H-HR-MAS NMR analysis of TrxR, TrxR DN, WT1, NR1, NR2, NR3, GusA, and WT2 trophozoites. The strains are detailed in Table 1. A, PCA; B, oPLS-DA, Figure S2: Metabolites identified in *G. lamblia* WBC6 wildtype trophozoites (WT, *n* = 4) and in trophozoites of the nitro drug-resistant strain C4 (*n* = 10); mean values ± SD. Metabolites marked with ** and * were significantly different with *p* < 0.001 and *p* < 0.005 respectively according to t-test and correction for multiple comparisons. Significant levels in gray were excluded from discussion due to high variability between different WT-batches, Figure S3. Representative ^1^H HR-MAS NMR spectra of *Giardia* trophozoites indicating selection of spectral regions / buckets and their metabolite assignments that were used for integration in univariate analysis. Ac (acetate) and Ile are better visible in the upper spectrum (A) than in the lower spectrum (A’). For metabolites marked with * the average of multiple regions as indicated were used. A-D, contiguous spectral regions; Cyn, cysteine, Table S1: Peak integrals obtained by ^1^H-HR-MAS NMR analysis of *G. lamblia* WBC6 wildtype trophozoites (WT; two independent experiments), in trophozoites overexpressing thioredoxin reductase (TrxR) or a dominant negative mutation of TrxR (TrxR DN) as a control, and in trophozoites overexpressing the nitroreductases NR1, NR2, NR3, or GusA as a control, Table S2: Summary of parameters and statistical results of the oPLS-DA shown in Figure 2, Figure 3 and Figure 5. Table S3: Peak integrals obtained by ^1^H-HR-MAS NMR analysis of *G. lamblia* WBC6 wildtype and nitro drug-resistant C4 trophozoites.
