# Supplementary material for: Metabolomic Profiling of Wildtype and Transgenic Giardia lamblia Strains by 1H HR-MAS NMR Spectroscopy
Source: Metabolites. 2020 Jan 30;10(2):53. doi: 10.3390/metabo10020053 (PMC7073884; doi:10.3390/metabo10020053)
Supplement: Supplementary file 1 [file metabolites-10-00053-s001.pdf]

Supplemental data

Table S1: Peak integrals obtained by ^{1}H-HR-MAS NMR analysis of *G. lamblia* WBC6 wildtype trophozoites (WT; two independent experiments), in trophozoites overexpressing thioredoxin reductase (TrxR) or a dominant negative mutation of TrxR (TrxR DN) as a control, and in trophozoites overexpressing the nitroreductases NR1, NR2, NR3, or GusA as a control.

	Normalized integrals [au]																										
	Leu	Val	Ile	Thre	Lys	Ala	Ac	Pip	Glu	Pro	Gln	Met	Citrate	Asn	Orn	Cys	Cys-cys	Ctl	TMA	Gly	G1P	Tyr	His	Phe	Trp	NAD	ATP
**WT 1-01**	4.31	4.28	0.46	2.97	4.77	5.21	0.77	0.68	7.12	0.57	0.65	1.05	0.21	0.19	1.86	0.58	0.06	0.14	1.10	2.05	4.83	0.39	0.12	0.29	0.08	0.07	0.25
**WT 1-02**	4.04	3.79	0.27	2.75	4.96	4.53	0.66	0.69	6.44	0.42	0.35	0.85	0.21	0.16	1.58	0.42	0.14	0.12	1.18	2.01	5.87	0.38	0.22	0.32	0.13	0.07	0.22
**WT 1-03**	4.45	4.27	0.55	3.03	5.16	5.88	0.77	0.79	7.11	0.44	0.42	0.90	0.15	0.18	1.73	0.47	0.13	0.20	1.25	2.17	3.69	0.35	0.18	0.31	0.12	0.09	0.28
**WT 1-04**	4.14	4.14	0.51	2.92	4.51	5.77	0.77	0.58	6.61	0.44	0.44	0.94	0.24	0.21	1.77	0.49	0.07	0.11	1.21	2.08	3.97	0.42	0.22	0.34	0.11	0.13	0.28
**WT 1-05**	4.15	4.19	0.12	2.81	4.55	6.01	0.78	0.67	7.04	0.49	0.54	0.92	0.09	0.17	1.86	0.60	0.15	0.26	1.17	2.21	4.09	0.44	0.18	0.31	0.13	0.08	0.27
**WT 1-06**	4.37	4.35	0.31	2.90	4.39	6.82	0.72	0.56	7.07	0.74	0.66	1.12	0.21	0.16	1.89	0.45	0.07	0.09	1.21	2.30	4.63	0.39	0.18	0.30	0.08	0.11	0.25
**WT 1-07**	4.63	4.54	0.37	2.96	4.72	7.21	0.77	0.55	7.25	0.69	0.44	0.98	0.06	0.16	2.01	0.59	0.10	0.17	1.19	2.30	3.72	0.38	0.22	0.26	0.11	0.06	0.28
**WT 1-08**	4.24	4.23	0.35	3.09	5.05	5.94	0.85	0.71	7.28	0.58	0.41	0.89	0.05	0.15	1.85	0.54	0.10	0.13	1.15	2.18	4.84	0.31	0.10	0.28	0.11	0.04	0.18
**WT 1-09**	4.27	4.35	0.75	3.10	4.70	6.31	0.82	0.58	7.09	0.49	0.30	0.87	0.22	0.22	2.01	0.83	0.17	0.40	1.14	2.25	4.36	0.35	0.23	0.30	0.05	0.07	0.25
**WT 1-10**	4.09	4.26	0.52	3.09	3.90	6.54	0.78	0.64	7.25	0.71	0.58	0.95	0.23	0.17	1.88	0.61	0.17	0.25	1.19	2.19	4.61	0.38	0.21	0.30	0.13	0.12	0.22
**WT 2-01**	5.79	5.96	0.69	2.70	4.21	7.47	0.84	0.87	5.40	0.71	1.08	0.97	0.21	0.29	1.67	0.32	0.13	0.37	1.10	2.82	1.70	0.40	0.30	0.34	0.07	0.08	0.21
**WT 2-02**	5.84	5.85	0.54	2.55	4.27	7.49	0.86	0.69	4.79	0.34	0.60	1.03	0.19	0.28	1.69	0.27	0.11	0.24	1.07	2.81	1.56	0.35	0.26	0.34	0.08	0.06	0.23
**WT 2-03**	5.90	5.79	0.88	2.55	4.07	7.41	0.89	0.60	4.74	0.31	0.64	1.14	0.19	0.25	1.73	0.37	0.17	0.28	1.09	2.88	1.59	0.38	0.26	0.33	0.11	0.05	0.23
**WT 2-04**	6.02	5.80	0.59	2.65	4.51	7.49	0.83	0.61	4.86	0.22	0.56	1.10	0.20	0.23	1.74	0.20	0.12	0.16	1.10	2.86	1.77	0.35	0.29	0.30	0.03	0.06	0.19
**TrxR-01**	3.72	4.68	0.53	3.43	5.75	4.52	0.57	0.68	7.40	0.38	0.24	0.93	0.15	0.15	1.70	0.21	0.11	0.00	1.21	2.31	4.73	0.41	0.21	0.31	0.10	0.08	0.33
**TrxR-02**	3.91	4.76	0.44	3.28	5.57	4.22	0.61	0.85	7.32	0.45	0.40	0.98	0.09	0.14	1.84	0.34	0.11	0.07	1.15	2.41	4.41	0.35	0.11	0.30	0.07	0.11	0.24
**TrxR-03**	3.93	4.88	0.30	3.26	5.30	4.93	0.53	0.63	7.35	0.52	0.52	1.09	0.26	0.18	2.11	0.63	0.18	0.35	1.17	2.31	4.27	0.33	0.15	0.28	0.07	0.07	0.33
**TrxR-04**	3.72	4.36	0.22	3.08	5.48	3.70	0.57	0.87	6.87	0.39	0.40	0.98	0.13	0.24	1.92	0.52	0.08	0.09	1.04	2.25	5.05	0.38	0.13	0.29	0.08	0.06	0.23
**TrxR-05**	3.83	4.53	0.43	3.12	5.76	3.77	0.55	0.72	7.11	0.45	0.57	1.02	0.13	0.18	1.91	0.52	0.04	0.06	1.15	2.34	4.67	0.38	0.09	0.31	0.13	0.08	0.25
**TrxR-06**	3.85	4.68	0.24	3.28	5.20	5.55	0.57	0.74	7.21	0.48	0.33	1.09	0.30	0.25	1.89	0.33	0.13	0.22	1.19	2.44	4.21	0.38	0.20	0.25	0.11	0.08	0.30
**TrxR-07**	4.01	4.72	0.29	3.29	5.70	5.02	0.60	0.73	7.33	0.51	0.44	1.03	0.09	0.15	1.92	0.36	0.12	0.15	1.17	2.39	4.94	0.36	0.12	0.25	0.07	0.07	0.24
**TrxR-08**	3.93	4.65	0.42	3.20	5.21	5.03	0.54	0.72	7.08	0.47	0.34	0.98	0.30	0.21	1.82	0.30	0.06	0.09	1.12	2.35	5.15	0.39	0.13	0.25	0.07	0.09	0.23

**TrxR-09**	4.26	5.11	0.31	3.47	5.38	6.62	0.59	0.70	7.80	0.82	0.74	1.04	0.26	0.18	1.96	0.32	0.16	0.16	1.22	2.55	3.89	0.34	0.08	0.26	0.11	0.09	0.34
**TrxR-10**	4.04	4.81	0.33	3.34	5.12	5.40	0.56	0.67	7.24	0.50	0.52	1.04	0.07	0.21	1.99	0.43	0.17	0.21	1.23	2.48	4.59	0.39	0.12	0.29	0.09	0.07	0.25
**TrxR DN-01**	3.71	4.31	0.26	2.82	5.20	4.33	0.65	0.86	6.86	0.38	0.49	0.99	0.20	0.19	1.57	0.28	0.20	0.14	1.19	2.21	5.74	0.34	0.17	0.33	0.13	0.09	0.26
**TrxR DN-02**	3.70	4.34	0.52	3.03	5.17	4.49	0.66	0.73	7.12	0.52	0.53	0.96	0.20	0.18	1.76	0.33	0.16	0.14	1.14	2.21	6.11	0.34	0.14	0.29	0.14	0.11	0.21
**TrxR DN-03**	3.66	4.37	0.29	3.00	4.85	5.17	0.69	0.78	7.13	0.47	0.42	1.01	0.33	0.25	1.65	0.32	0.19	0.18	1.17	2.13	4.58	0.34	0.22	0.29	0.12	0.07	0.27
**TrxR DN-04**	3.88	4.67	0.33	2.94	5.08	5.17	0.58	0.62	7.15	0.32	0.41	1.03	0.29	0.19	1.57	0.26	0.08	0.18	1.11	2.20	4.98	0.34	0.12	0.23	0.10	0.06	0.22
**TrxR DN-05**	3.43	4.00	0.37	2.83	5.03	4.10	0.60	0.90	6.61	0.45	0.71	1.04	0.35	0.23	1.54	0.40	0.10	0.31	1.04	1.95	5.05	0.33	0.20	0.31	0.07	0.10	0.28
**TrxR DN-06**	3.75	4.36	0.34	3.12	5.54	5.35	0.69	0.72	7.33	0.42	0.43	1.02	0.16	0.23	1.72	0.38	0.16	0.15	1.25	2.21	4.63	0.33	0.09	0.24	0.12	0.04	0.24
**TrxR DN-07**	3.72	4.44	0.26	3.22	5.02	5.17	0.65	0.73	7.19	0.63	0.50	0.90	0.24	0.24	1.80	0.35	0.11	0.18	1.14	2.31	6.25	0.33	0.12	0.30	0.08	0.12	0.19
**TrxR DN-08**	4.08	4.92	0.44	3.22	4.83	6.75	0.63	0.66	7.34	0.68	0.41	0.91	0.23	0.23	1.92	0.33	0.14	0.16	1.24	2.34	4.52	0.34	0.16	0.22	0.04	0.08	0.23
**TrxR DN-09**	3.75	4.48	0.47	3.04	4.72	5.56	0.67	0.50	7.05	0.57	0.50	0.98	0.23	0.23	1.83	0.33	0.15	0.17	1.19	2.26	5.20	0.41	0.16	0.28	0.10	0.09	0.28
**TrxR DN-10**	3.91	4.73	0.24	3.10	4.79	5.95	0.69	0.71	7.45	0.59	0.42	0.98	0.18	0.19	1.83	0.31	0.17	0.18	1.16	2.33	4.52	0.33	0.19	0.27	0.08	0.05	0.29

Table S1 continued: Peak integrals obtained by ^{1}H-HR-MAS NMR analysis of *G. lamblia* WBC6 wildtype trophozoites (WT; two independent experiments), in trophozoites overexpressing thioredoxin reductase (TrxR) or a dominant negative mutation of TrxR (TrxR DN) as a control, and in trophozoites overexpressing the nitroreductases NR1, NR2, NR3, or GusA as a control.

	Normalized integrals [au]																										
	Leu	Val	Ile	Thre	Lys	Ala	Ac	Pip	Glu	Pro	Gln	Met	Citrate	Asn	Orn	Cys	Cys-cys	Ctl	TMA	Gly	G1P	Tyr	His	Phe	Trp	NAD	ATP
**NR1-01**	3.84	5.07	0.70	2.48	4.38	5.08	0.48	1.22	5.22	0.58	0.49	1.33	0.25	0.13	2.45	0.42	0.49	1.35	0.76	2.35	2.17	0.25	0.14	0.17	0.02	0.06	0.26
**NR1-02**	3.99	5.40	0.69	2.54	4.43	5.66	0.53	1.25	5.52	0.49	0.28	1.25	0.22	0.10	2.90	0.45	0.42	1.18	0.70	2.53	2.29	0.24	0.05	0.15	0.01	0.05	0.24
**NR1-03**	4.26	5.54	0.69	2.59	4.56	5.65	0.50	1.14	5.48	0.58	0.32	1.34	0.46	0.22	2.62	0.26	0.44	1.08	0.73	2.49	2.47	0.26	0.16	0.23	0.03	0.06	0.28
**NR1-04**	4.25	5.16	0.61	2.41	3.91	5.31	0.47	1.30	5.31	0.57	0.38	1.26	0.28	0.17	2.71	0.38	0.52	0.98	0.70	2.27	2.41	0.28	0.17	0.26	0.04	0.10	0.24
**NR1-05**	4.09	5.17	0.70	2.47	4.39	5.31	0.48	1.23	5.22	0.48	0.26	1.29	0.37	0.27	2.56	0.40	0.47	1.24	0.68	2.36	2.21	0.27	0.16	0.18	0.03	0.06	0.28
**NR2-01**	5.63	6.23	0.74	2.66	3.68	6.44	0.59	1.13	5.31	0.74	0.85	1.24	0.13	0.19	1.87	0.20	0.47	0.50	0.93	2.67	1.36	0.35	0.24	0.29	0.09	0.11	0.36
**NR2-02**	5.28	5.90	0.79	2.69	4.05	6.07	0.55	1.13	5.22	0.69	0.66	1.14	0.14	0.24	1.96	0.33	0.41	0.43	0.89	2.64	1.73	0.27	0.19	0.24	0.04	0.10	0.33
**NR2-03**	5.48	5.89	1.00	2.94	4.25	6.03	0.57	1.07	5.02	0.51	0.48	1.13	0.11	0.22	2.08	0.48	0.46	0.61	0.88	2.62	1.82	0.25	0.21	0.26	0.09	0.05	0.31
**NR2-04**	5.61	5.59	0.95	2.92	4.22	5.18	0.59	1.07	5.10	0.83	1.06	1.13	0.24	0.18	1.75	0.08	0.33	0.16	0.72	2.63	1.99	0.43	0.25	0.26	0.13	0.05	0.24
**NR2-05**	5.59	6.11	0.90	2.94	4.21	6.26	0.59	0.96	5.05	0.43	0.23	1.14	0.30	0.24	1.95	0.36	0.44	0.61	0.91	2.69	1.62	0.30	0.20	0.19	0.04	0.08	0.26
**NR2-06**	5.07	5.77	0.85	2.76	4.30	5.93	0.55	1.41	5.24	0.75	0.83	1.16	0.28	0.23	2.01	0.52	0.46	0.68	0.94	2.57	1.59	0.27	0.25	0.26	0.04	0.02	0.31
**NR3-01**	5.24	6.08	0.79	3.04	4.79	7.06	0.62	1.06	5.58	0.72	1.03	1.15	0.13	0.24	1.82	0.26	0.26	0.23	1.00	3.05	1.78	0.37	0.19	0.30	0.01	0.08	0.23
**NR3-02**	5.29	6.11	0.69	3.06	4.66	7.28	0.58	0.99	5.60	0.72	0.99	1.07	0.11	0.26	1.83	0.27	0.26	0.24	1.07	2.98	1.83	0.34	0.25	0.28	0.04	0.08	0.23
**NR3-03**	5.10	5.89	0.67	2.95	4.79	6.44	0.57	1.07	5.50	0.74	0.92	1.07	0.14	0.25	1.82	0.33	0.25	0.19	1.03	2.94	2.13	0.35	0.20	0.26	0.05	0.10	0.27
**NR3-04**	5.00	5.72	0.82	2.90	4.38	6.52	0.57	1.06	5.20	0.60	0.80	1.02	0.20	0.29	1.90	0.50	0.34	0.44	1.06	2.86	1.85	0.34	0.21	0.28	0.05	0.06	0.25
**NR3-05**	5.32	5.87	0.86	3.05	4.80	6.68	0.57	0.83	5.20	0.53	0.80	1.13	0.21	0.26	1.68	0.11	0.22	0.08	1.02	2.97	1.82	0.39	0.27	0.30	0.07	0.09	0.25
**GusA-01**	5.90	5.64	0.85	2.64	4.26	6.44	0.65	1.04	5.15	0.64	0.84	0.95	0.09	0.24	1.70	0.37	0.32	0.33	1.14	2.76	1.44	0.33	0.29	0.29	0.06	0.12	0.24
**GusA-02**	5.92	5.89	0.84	2.91	4.45	7.19	0.66	0.95	5.25	0.56	0.83	1.07	0.13	0.23	1.64	0.22	0.23	0.33	1.12	2.89	1.55	0.36	0.24	0.29	0.03	0.08	0.25
**GusA-03**	6.01	5.86	0.64	2.67	4.35	6.94	0.66	1.02	5.30	0.73	0.98	1.01	0.06	0.22	1.67	0.15	0.22	0.18	1.09	2.82	1.91	0.36	0.25	0.27	0.04	0.05	0.22
**GusA-04**	6.01	5.97	0.60	2.78	4.76	7.06	0.61	1.03	5.54	0.74	1.06	1.08	0.07	0.22	1.65	0.18	0.19	0.20	1.09	2.89	1.76	0.37	0.27	0.30	0.08	0.08	0.25
**GusA-05**	5.89	5.97	0.69	2.76	4.02	7.14	0.68	0.97	5.38	0.72	0.97	0.99	0.17	0.19	1.65	0.18	0.25	0.26	1.15	2.91	1.77	0.37	0.27	0.27	0.06	0.03	0.23
**GusA-06**	5.63	5.68	0.78	2.68	4.12	6.82	0.64	0.86	5.15	0.62	0.80	1.03	0.18	0.27	1.75	0.45	0.25	0.31	1.10	2.85	1.62	0.41	0.27	0.35	0.07	0.08	0.25

Table S2. Summary of parameters and statistical results of the oPLS-DA shown in Figures 2, 3 and 5.

**Groups**	**Fig. 2**	**Fig. 3**	**Fig. 5**
Preprocessing *	PQN, mean center, Pareto scaling		
Cross validation	Venetian blinds 5 splits, 1 sample/split		
X-block	20 × 138	22 x 138	14 × 138
Number of LVs	2	3	1
LV 1	22.98%	37.21%	69.6%
LV 2	28.17%	27.18%	-
LV 3	-	5.55%	-
Total variance captured	51.15%	69.94%	69.6%
Modeled class	1 / 2	1 / 2 / 3	1 / 2
R^2	0.878 / 0.878	0.928 / 0.797 / 0.908	
Q^2	0.731 / 0.731	0.776 / 0.589 / 0.625	

PQN: Probabilistic quotient normalization [48]; LV: Latent variable; R^2: R^2 calculated; Q^2: R^2 cross-validated

Table S3: Peak integrals obtained by ^{1}H-HR-MAS NMR analysis of *G. lamblia* WBC6 wildtype and nitro drug-resistant C4 trophozoites.

	Normalized integrals [au]																										
	Leu	Val	Ile	Thre	Lys	Ala	Ac	Pip	Glu	Pro	Gln	Met	Citrate	Asn	Orn	Cys	Cys-Cys	Ctl	TMA	Gly	G1P	Tyr	His	Phe	Trp	NAD	ATP
**C4-01**	4.38	4.54	0.51	3.51	5.58	3.82	0.68	0.89	5.15	0.31	0.30	1.01	0.27	0.30	1.39	0.94	0.18	0.21	0.96	2.37	4.56	0.35	0.08	0.29	0.08	0.04	0.17
**C4-02**	4.60	4.78	0.59	3.66	5.70	4.11	0.73	0.99	5.37	0.23	0.28	1.03	0.31	0.29	1.48	1.07	0.12	0.20	0.89	2.39	3.91	0.41	0.13	0.29	0.08	0.07	0.18
**C4-03**	5.16	5.36	0.70	3.95	6.32	4.74	0.73	1.02	5.85	0.32	0.49	1.12	0.18	0.26	1.44	0.95	0.15	0.12	1.11	2.62	3.48	0.41	0.14	0.35	0.12	0.12	0.23
**C4-04**	4.85	4.98	0.68	3.85	5.98	4.53	0.77	0.95	5.56	0.26	0.36	1.14	0.22	0.27	1.42	0.96	0.14	0.10	1.05	2.57	3.68	0.41	0.09	0.34	0.11	0.06	0.25
**C4-05**	5.03	5.30	0.60	4.02	5.87	4.77	0.79	1.11	6.01	0.36	0.50	1.16	0.16	0.22	1.42	0.95	0.22	0.13	1.19	2.69	3.58	0.35	0.06	0.28	0.06	0.11	0.17
**C4-06**	4.71	5.01	0.59	3.78	5.65	4.79	0.75	1.11	5.53	0.35	0.62	1.12	0.25	0.28	1.44	1.08	0.21	0.24	1.03	2.46	2.77	0.39	0.10	0.33	0.09	0.09	0.25
**C4-07**	4.56	4.81	0.50	3.56	5.64	4.33	0.74	1.03	5.43	0.40	0.50	1.09	0.18	0.22	1.29	0.79	0.18	0.08	1.00	2.46	3.83	0.39	0.12	0.32	0.10	0.08	0.15
**C4-08**	4.69	5.14	0.68	3.90	5.81	4.73	0.76	1.09	5.70	0.37	0.41	1.07	0.24	0.24	1.39	0.86	0.17	0.08	1.09	2.57	3.47	0.34	0.06	0.32	0.08	0.08	0.21
**C4-09**	5.07	5.31	0.57	3.94	6.10	4.93	0.76	1.09	5.91	0.41	0.68	1.07	0.12	0.21	1.55	1.16	0.18	0.34	1.01	2.59	2.79	0.40	0.11	0.36	0.08	0.10	0.24
**C4-10**	4.67	4.91	0.62	3.60	5.25	4.37	0.68	0.74	5.26	0.29	0.42	1.08	0.24	0.24	1.47	1.07	0.21	0.28	0.99	2.38	4.23	0.33	0.04	0.28	0.07	0.08	0.13
**WT-01**	1.97	3.76	0.43	1.86	3.41	5.06	0.35	0.83	7.19	0.54	0.27	1.25	0.36	0.17	4.38	1.43	0.30	4.41	0.46	1.56	3.92	0.26	0.05	0.15	0.12	0.12	0.37
**WT-02**	1.97	4.27	0.72	2.35	3.32	5.94	0.29	0.67	7.62	0.49	0.00	1.16	0.35	0.11	4.44	1.33	0.37	4.88	0.72	1.77	4.08	0.27	0.04	0.09	0.09	0.14	0.40
**WT-03**	2.05	4.36	0.62	2.13	2.84	5.99	0.38	0.80	7.79	0.53	0.19	1.41	0.62	0.16	3.92	0.82	0.23	5.05	0.78	1.79	3.81	0.28	0.18	0.13	0.05	0.11	0.36
**WT-04**	1.90	4.30	0.85	2.34	2.97	5.92	0.34	0.83	8.00	0.63	0.12	1.28	0.58	0.23	3.88	0.81	0.24	5.10	0.72	1.73	3.96	0.27	0.05	0.17	0.13	0.10	0.41

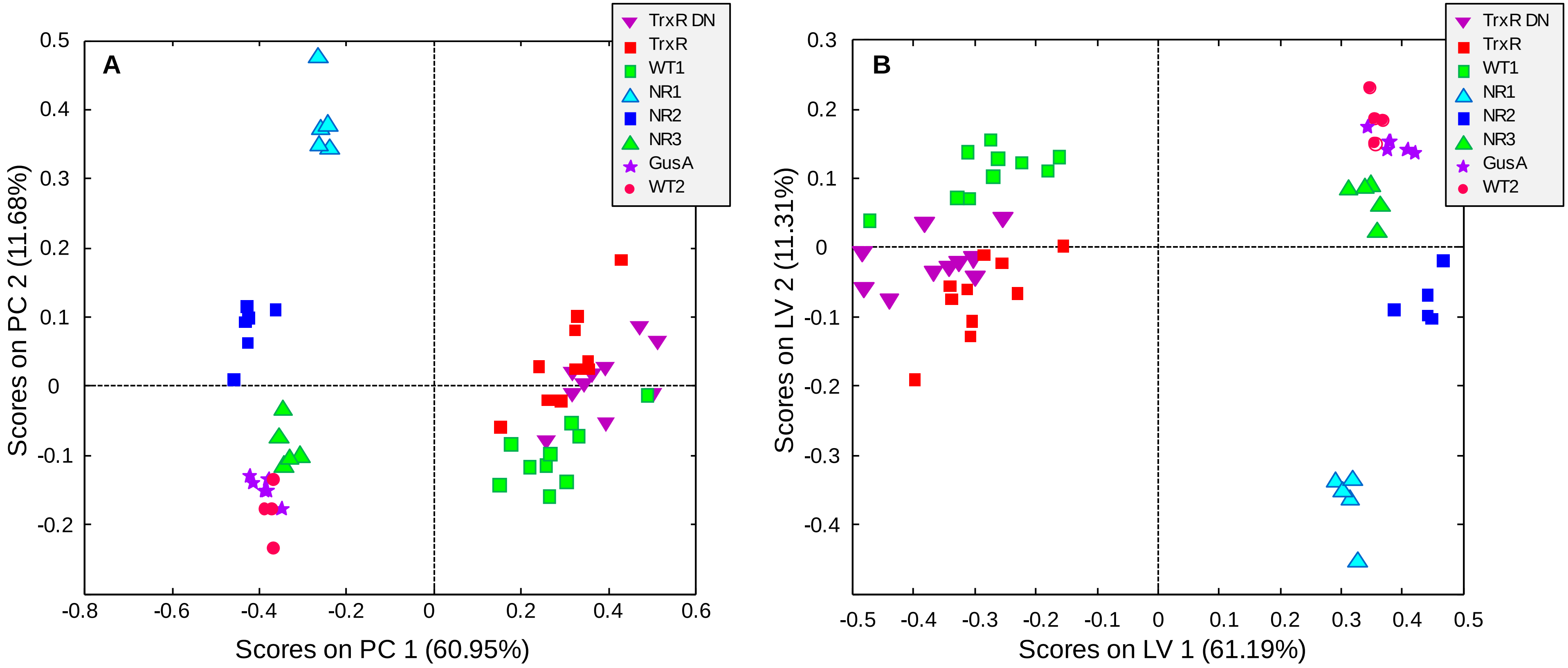

Figure S1. PCA and oPLS-DA of all integral regions (138 buckets) obtained by ^{1}H-HR-MAS NMR analysis of TrxR, TrxR DN, WT1, NR1, NR2, NR3, GusA, and WT2 trophozoites. The strains are detailed in Table 1. A, PCA; B, oPLS-DA.

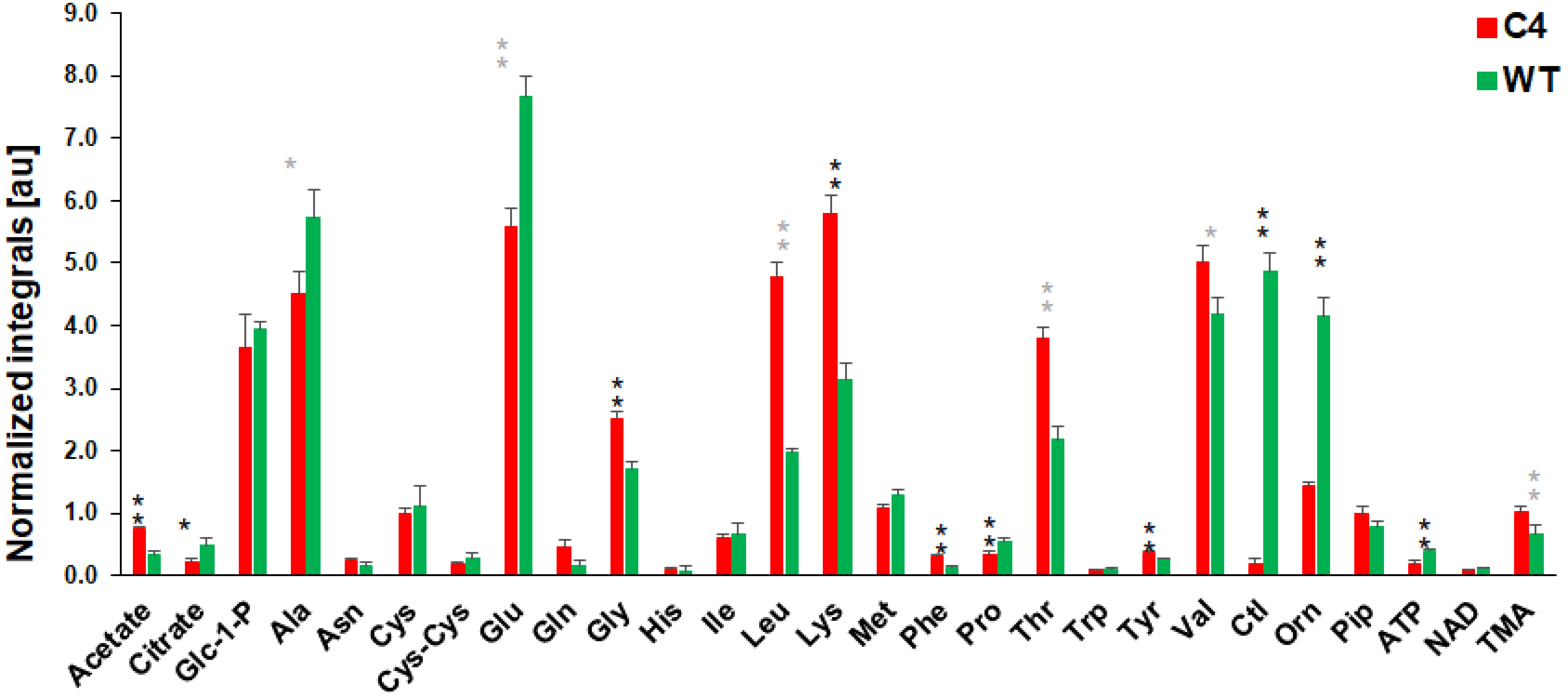

Figure S2. Metabolites identified in *G. lamblia* WBC6 wildtype trophozoites (WT, n = 4) and in trophozoites of the nitro drug-resistant strain C4 (n = 10); mean values ± SD. Metabolites marked with ** and * were significantly different with $p < 0.001$ and $p < 0.005$ respectively according to t-test and correction for multiple comparisons. Significant levels in gray were excluded from discussion due to high variability between different WT-batches. The ^{1}H-HR-MAS NMR analysis has been performed as described in Materials and Methods.

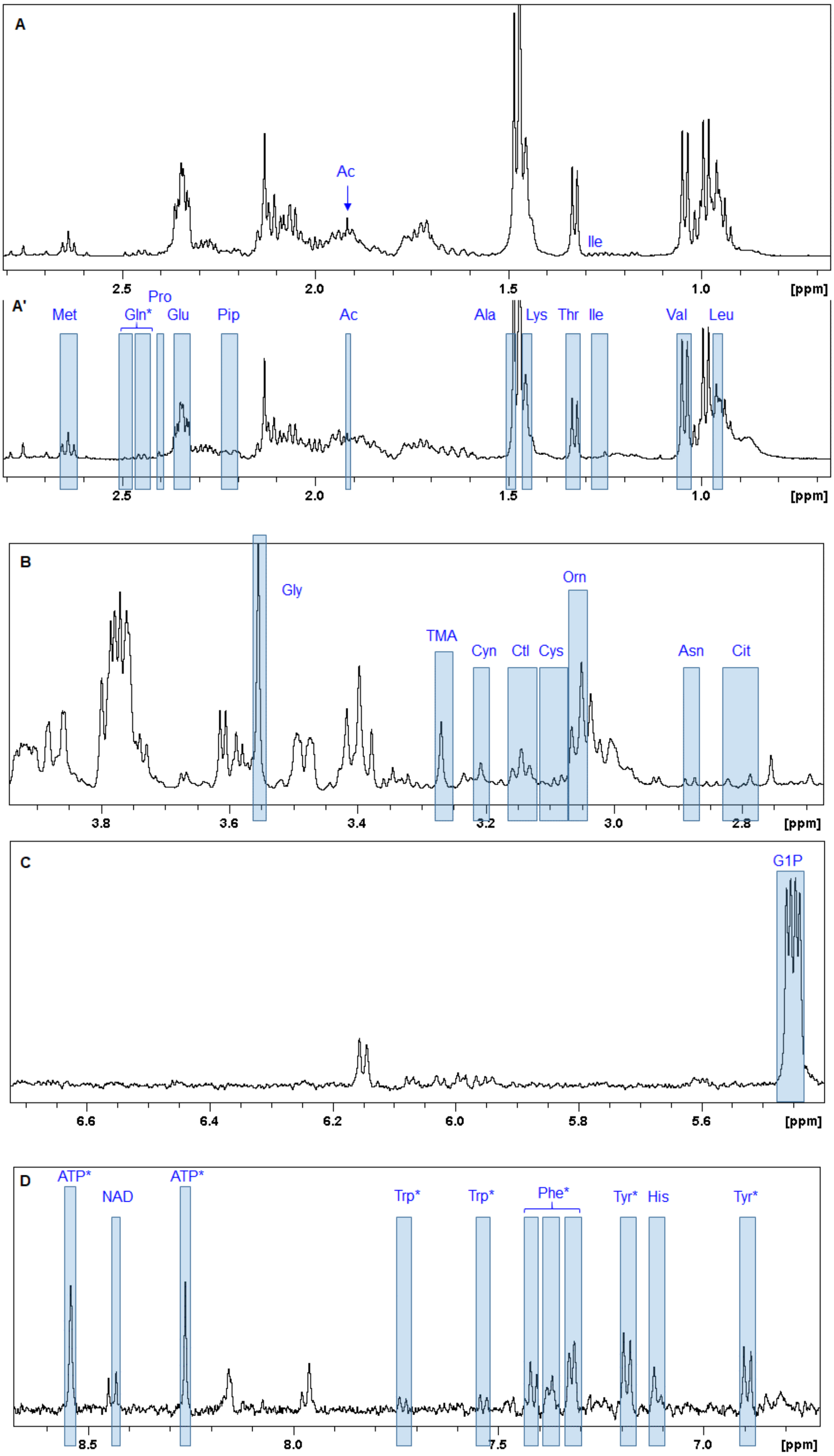

Figure S3. Representative ¹H HR-MAS NMR spectra of *Giardia* trophozoites indicating selection of spectral regions / buckets and their metabolite assignments that were used for integration in univariate analysis. Ac (acetate) and Ile are better visible in the upper spectrum (A) than in the lower spectrum (A′). For metabolites marked with * the average of multiple regions as indicated were used. A-D, contiguous spectral regions; Cyn, cystine.
